# *In vivo* imaging of CREB phosphorylation in awake-mouse brain

**DOI:** 10.1038/srep09757

**Published:** 2015-06-05

**Authors:** Tetsuya Ishimoto, Hiroki Mano, Hisashi Mori

**Affiliations:** 1 Department of Molecular Neuroscience, Graduate School of Medicine and Pharmaceutical Sciences, University of Toyama, 2630 Sugitani, Toyama 930-0194, Japan

## Abstract

The cyclic adenosine monophosphate response element binding protein (CREB) is a phosphorylation-dependent transcription factor that plays important roles in memory consolidation and several neuropsychological disorders. Although analyzing the spatiotemporal pattern of CREB phosphorylation is required for elucidating the mechanism of memory consolidation, imaging of phosphorylation of a particular protein in the brain of live animals is impossible at present. Here, we developed a method for visualizing the CREB phosphorylation in the cerebral cortex of an awake mouse using a split luciferase technique. Using this technique, we demonstrated the correlation between the change in CREB phosphorylation at a particular region in the brain and behavioral consequences induced by the administration of reserpine, a psychotropic agent.

I*n vivo* imaging technologies have great potentials for elucidating the molecular basis of physiological and pathological events inside live animals. PET^1^ and MRI^2^ have been used for analyzing molecular localization in and morphology of human and rodent brains. Fluorescent compounds^3^,^4^,^5^ have been used for monitoring Ca^2+^ signals in neurons by *in vivo* two-photon microscopy. Firefly luciferase can be a transcription monitor when it is expressed in animals with the promoter of a particular gene^6^,^7^,^8^. Despite the advances in these imaging technologies, imaging of modifications of a particular protein such as phosphorylation in the brain of live animals is not possible at present.

CREB is a transcription factor that was first discovered as a DNA-binding protein that recognizes the cAMP response element (CRE) (TGACGTCA)^9^,^10^. Once CREB is phosphorylated at serine 133, it interacts with the CREB-binding protein (CBP) or its paralogue p300 to up-regulate transcriptional activity^11^. The binding domains of CREB and CBP have been identified as the kinase inducible domain (KID) and KIX, respectively^11^. CREB-mediated transcriptional up-regulation is considered to play important roles in memory consolidation^12^,^13^,^14^. On the other hand, overphosphorylation of CREB is observed in addiction^15^,^16^, and CREB activation in the nucleus accumbens results in resistance to extinction of fear memory^17^. In patients with major depressive disorder and mouse depression models, both the up-regulation^18^,^19^ and down-regulation^20^,^21^ of CREB phosphorylation were reported. Thus, measuring CREB phosphorylation level in the brain of live animals is required for elucidating the mechanism of memory consolidation and neuropsychological disorder.

In our previous study, we succeeded in developing bioluminescence probes^22^ for detecting CREB phosphorylation in HEK293T cells on the basis of a split luciferase technique^23^,^24^,^25^. The probes consist of nuclear localization signal attached KID-C-terminal luciferase (KID-lucC) and N-terminal luciferase-KIX (lucN-KIX)^22^. The interaction between KID and KIX leads to complementation of lucN and lucC, restoration of luciferase activity, and subsequent photon emission. It is confirmed that KID-KIX interaction and subsequent increase in light emission were dependent on serine 133 phosphorylation in the KID^22^. In current study, we generated a transgenic (Tg) mouse line that expressed these probes and detected light emission from the brain of an awake mouse. Furthermore, we determined the correlation between CREB phosphorylation in a particular region in the cerebral cortex and depression-like symptoms induced by the psychotropic agent reserpine.

## Results

### Changes in light intensity in cerebral cortex of Tg mice induced by acute imipramine treatment

**Figure 1:**
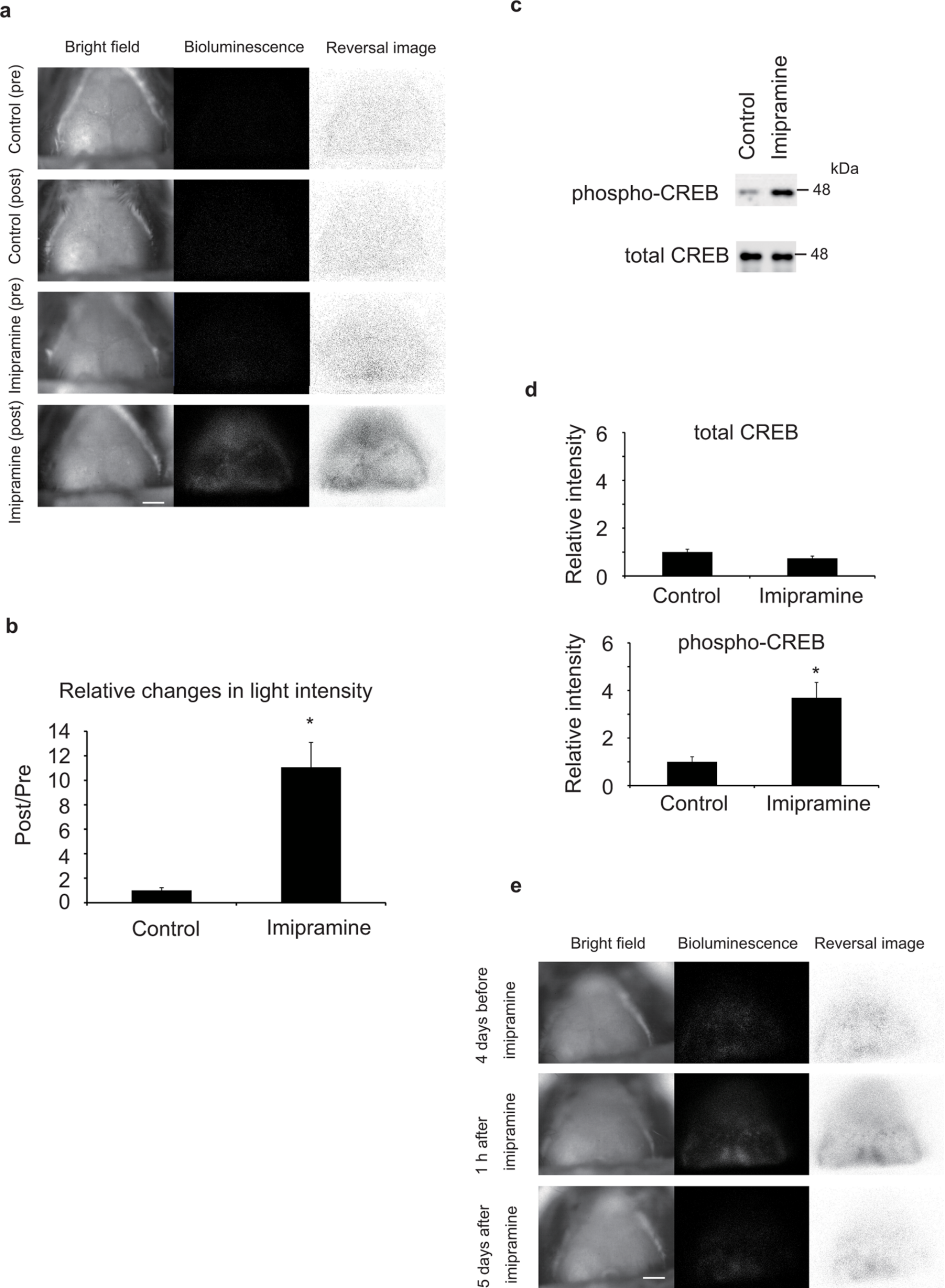
Increase in light emission intensity and CREB phosphorylation in acute-imipramine-treated mice. (a) Bright-field images (left) and bioluminescence images of light emission from cerebral cortex (center) before and after imipramine treatment. Bioluminescence images in which black and white were reversed and contrasts were adjusted for visibility are shown on the right. Bar indicates 2 mm. (b) Relative changes in light emission intensity before and after imipramine treatment. Light emission from the cerebral cortex is quantified. Data are presented as mean ± SEM, n = 4. * *p* < 0.05, with Student's *t*-test. (c) Analysis of CREB expression by western blotting. The cerebral cortex of the mice treated with imipramine (64 mg/kg) for 1 h was analyzed using anti-phospho-CREB and anti-CREB antibodies. The position of the size marker (48 kDa) is indicated on the right. (d) Changes in phospho-CREB expression level after treatment of imipramine. The bands in the western blotting were quantified. The vertical axis represents relative intensity that was normalized to the controls. Data are presented as mean ± SEM, n = 4. * *p* < 0.05, with Student's *t*-test. (e) An example of repetitive imaging of CREB phosphorylation in live mouse brain. Images were taken at 3 time points. Bar indicates 2 mm.

The Tg mouse line that expressed probe proteins (lucN-KIX and KID-lucC) driven by the β-actin promoter was successfully generated (see Supplementary Fig. S1a, b). Then, we determined whether the change in the intensity of light emission from the cerebral cortex was accompanied by endogenous CREB phosphorylation. The Tg mouse was injected with imipramine (64 mg/kg body weight), a tricyclic antidepressant that up-regulates CREB phosphorylation^26^, and the light emitted from the mouse brain was imaged 1 h after injection using a newly designed head fixation technique (see Supplementary Fig. S2a, b). We found an increased light emission intensity in the cerebral region of the imipramine-treated mouse brain (Fig. 1a, b). Because it is considered that the main target of imipramine is central neurons *in vivo*^27^ and the probe proteins are driven by housekeeping β actin promoter, the increase in light emission intensity reflects the increase in the intensity of light emission from the probes expressed in the neurons of the cerebral cortex. The level of phospho-CREB was also observed to increase in the imipramine-treated cerebral cortex by western blotting (Fig. 1c, d). An example of repetitive imaging before and after imipramine treatment is shown in Figure 1e. Five days after the treatment, the increased light intensity seen at 1 h after imipramine treatment was disappeared. This result demonstrates that our method can be used repetitively, and indicates the effect of imipramine on CREB phosphorylation is temporal.

To determine the effect of fixation using metal plate and magnet on the stress level and CREB phosphorylation, we measured the concentration of corticosterone in the serum which was known to reflect the stress level. We found no increase in corticosterone concentration 5 min after fixation (see Supplementary Fig. S3a). We also found no increase in phospho CREB level in the cerebral cortex using western blotting (see Supplementary Fig. S3b). These results indicate the changes in light emission induced by imipramine (Fig. 1a) were not due to the stress induced by 5 min fixation.

The subcellular localization of probe proteins was analyzed by immunohistochemistry using the cortical slice of the transgenic mouse. We found the signal representing the luciferase protein was located at least in the nucleus of the neuron since the luciferase signal was merged with the signal of neuron-specific protein NeuN (see Supplementary Fig. S3c). Furthermore, to ensure that the changes in light emission reflects the CREB phosphorylation in neuron, we determined whether light emission from cultured neuron is up-regulated by forskolin which was known to activate adenylyl cyclase-protein kinase A-CREB pathway. The light intensity from transgenic cortical culture (DIV 3) which mainly consisted of neuron was measured after forskolin treatment. The forskolin-treated neuron showed significant increase in light intensity 1 h after the treatment (see Supplementary Fig. S3d).

### Changes in light emission pattern in mouse depression model induced by reserpine

**Figure 2:**
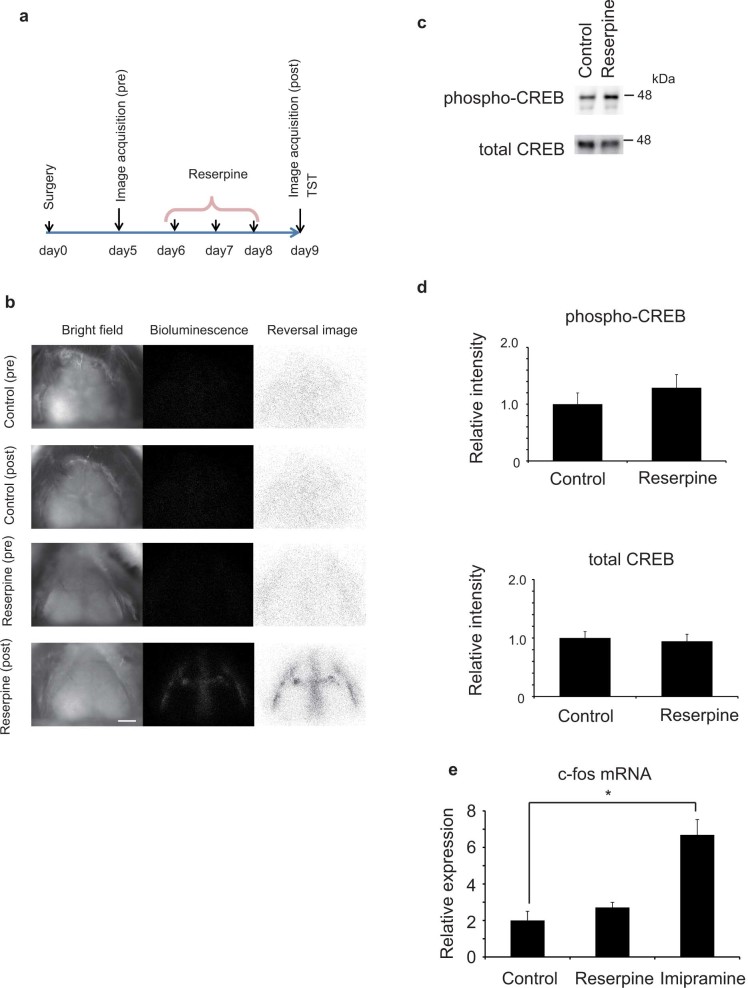
Changes in pattern of light emission induced by reserpine treatment. (a) Schedule of experiment. Mice were treated with reserpine for three consecutive days and then subjected to TST 24 h after the last treatment. Bioluminescence images of the cerebral cortex were taken before and after reserpine treatment. (b) Light emission from cerebral cortex of mice before and after reserpine treatment (1 mg/kg/day). The bright-field image and raw image of bioluminescent light emission are represented in the left and center columns, respectively. Reverse images with contrast adjustment are shown on the right. Bar indicates 2 mm. (c) Analysis of phosphorylated CREB and total CREB in cerebral cortex of reserpine-treated (1 mg/kg/day) mice by western blotting. The position of size marker (48 kDa) is indicated on the right. (d) Quantification of the bands for phosphorylated and total CREB in western blotting. Data are presented as mean ± SEM, n = 4. (e) Changes in the amount of mRNA for c-fos after reserpine (1 mg/kg/day) and imipramine (64 mg/kg) treatments. Total RNA from cerebral cortex after the stimuli are reverse transcribed and analyzed by quantitative PCR. Data are presented as mean ± SEM, n = 4. * *p* < 0.05, with Student's *t*-test. n = 4.

Next, we tried to evaluate whether this system can be used for analyzing the correlation between the CREB phosphorylation pattern in the cerebral cortex and behavioral changes induced by the psychotropic agent. There were some reports that indicated the up-regulation of CREB phosphorylation in animal depression models and patients with major depressive disorder in particular regions of the brain^18^,^19^. However, other studies showed down-regulation of CREB phosphorylation^20^,^21^. Thus, there is no consensus on whether CREB phosphorylation is accelerated under depressive condition, and it is worth examining the precise spatial CREB phosphorylation profile in the brain of a mouse depression model to elucidate the mechanism of development of the disorder. We determined whether the treatment with reserpine, which induces depression-like symptoms in mice^28^, changed the pattern of CREB phosphorylation in the mouse brain. Tg mice were injected with reserpine for three consecutive days at doses of 0, 0.5 and 1 mg/kg body weight. One day after the last reserpine treatment, light emission from mice brain was imaged and depression symptoms were measured using the tail suspension test (TST), which is a well-established test for measuring severity of depression-like symptoms^29^ (Fig. 2a). Some reserpine-treated mice showed a marked increase in the intensity of light emission from particular regions of the frontal cortex (Fig. 2b). On the other hand, control mice and some reserpine-treated mice showed no change in light emission pattern (Fig. 2b).

We confirmed by quantitative PCR that the mRNA expression levels of probe proteins did not change 3 days after reserpine (1 mg/kg body weight) treatment (see Supplementary Fig. S4). Thus, the changes in light emission pattern induced by reserpine were ascribed to the changes in the intensity of light emission from probe proteins and not to the changes in the expression level of the probe proteins. In western blotting, the increase in endogenous CREB phosphorylation level in the entire cerebral cortex of reserpine (1 mg/kg)-treated mice (Fig. 2c, d) was not significant. The expression of c-fos mRNA in the cerebral cortex after imipramine (64 mg/kg, 1 h) and reserpine (1 mg/kg/day, 3 days) treatment was determined using quantitative PCR. We found significant up-regulation of c-fos mRNA after imipramine treatment but not in reserpine-treated mice (Fig. 2e). This result reflects the changes in light intensity after treatment of imipramine and reserpine, and supports the reliability of this method.

### Correlation between light emission pattern and depression-like behavior in reserpine-treated mice

**Figure 3:**
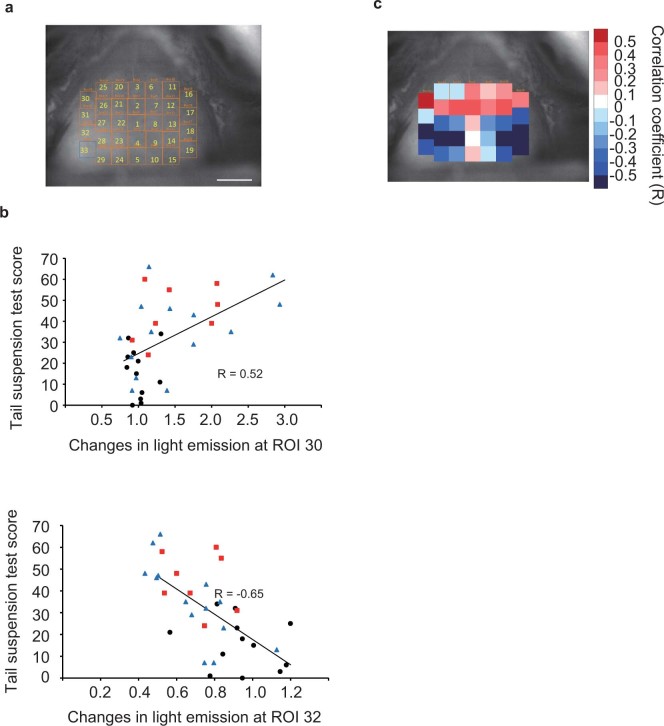
Analysis of correlation between changes in intensity of light emission from ROIs in the cerebral cortex and TST immobility score. (a) ROI setting on the brain. The first ROI (50 × 50 pixels) was set on the bregma of the brain. The locations of ROIs are indicated by numbers. Bar indicates 2 mm. (b) Scatter plot analysis of TST score and the ratio of increase in intensity of light emission from particular ROIs. Plots for ROIs 30 (upper) and 32 (lower), which exhibited positive and negative correlation coefficients (R), are shown as representatives. Black, red, and blue markers are the data from mice treated with reserpine at doses of 0, 0.5, and 1 mg/kg/day, respectively. Linear approximations and correlation coefficients (R) analyzed using all the dots are represented. (c) Pseudocolor representation of correlation coefficient (R) in all ROIs. Strong red ROIs indicate a high correlation between increase in light emission intensity and depressive symptoms.

After the imaging of light emission from the cerebral cortex, TST was performed to evaluate the severity of depression-like symptoms in each mouse. Subsequently, we tried to identify the region in which CREB was phosphorylated and examined its correlation with depression-like symptoms. We set 33 regions of interest (ROIs) on the cerebral cortex, and the rate of the changes in light emission intensity after reserpine treatment was calculated in each ROI (Fig. 3a). The scatter plots of the rate of change in particular ROIs from each mouse as a function of TST immobility score were drawn, and a linear line was fitted to the data and the correlation coefficient (R) was calculated (Fig. 3b). We found a positive correlation in ROI 30, that is, the increase in CREB phosphorylation level in ROI 30 was related to the severity of depression-like symptoms. On the other hand, ROI 32 showed an negative correlation (Fig. 3b). Other than ROIs 30 and 32, some ROIs showed high correlations with TST immobility score (see Supplementary Table S1). The calculated correlation coefficients are shown as pseudocolors on the bright-field brain images in Figure 3c, which shows positive and negative correlations depending on the region of the cerebral cortex.

## Discussion

We observed an increase in light emission intensity in awake mouse brain accompanied by elevated endogenous CREB phosphorylation levels in response to the acute administration of imipramine (Fig. 1a–d), a tricyclic antidepressant reported to up-regulate CREB phosphorylation^26^. This observation strongly suggests that the up-regulation of light emission from the mouse cerebral cortex reflects endogenous CREB phosphorylation, which is in agreement with our previous report that demonstrated phosphorylation-dependent up-regulation of light emission from the probe proteins that occurred in association with endogenous CREB phosphorylation *in vitro*^22^. We also confirmed the changes in light emission were not due to the increased stress level by fixation (see Supplementary Fig. S3a, b) and suggested the light was emitted at least from neuron (see Supplementary Fig. S3c, d). Because this method enables repetitive imaging of CREB phosphorylation (Fig. 1e), unlike conventional western blotting that requires sacrificing the mice, spatial and temporal changes of the phosphorylation can be measured in a mouse. Since CREB phosphorylation is related to memory consolidation and neuropsychological disorders, this imaging method is expected to elucidate their mechanisms by analyzing the spatiotemporal pattern of CREB phosphorylation. Moreover, it may be used for the screening of drugs that affect CREB phosphorylation *in vivo*. By generating Tg mice expressing other split luciferase probes designed to detect particular interactions, it can be a general method for imaging protein-protein interactions *in vivo*. Since we aimed to observe apparent change in light emission along with CREB phosphorylation in current study, higher dose (64 mg/kg) of imipramine was used in Fig. 1. Lower dose (10 mg/kg) treated mouse did not show statistically-significant increase in light intensity from mouse cortex (data not shown). It may be because the change in CREB phosphorylation with low dose of imipramine was smaller than high dose (64 mg/kg) treatment. In the future study, it will be required to determine the sensitivity and linearity to the level of endogenous CREB phosphorylation.

We used a mouse depression model induced by reserpine treatment (Fig. 2a–d). Reserpine induces not only depression-like symptoms but also symptoms of other diseases such as Parkinson's disease^30^. Thus, the changes in light emission intensity induced by reserpine are not necessarily related to depression; thus we need to confirm whether the changes correlate with the symptoms of depression. Analysis of correlation between changes in light emission intensity in each ROI and TST immobility score identified a region (ROI 30) that positively correlated (Fig. 3a–c), indicating that an elevated CREB phosphorylation level in ROI 30 is related to depression-like symptoms. Conversely, ROI 32 was found to have a negative correlation with TST immobility score. These ROIs are located at the border of the motor cortex and somatosensory cortex (ROI 30) and somatosensory cortex (ROI 32). To determine how the CREB phosphorylation in ROI 30 or 32 affects the development of depression-like symptoms, further research using other methods will be needed.

There are some reports indicating the up-regulation or down-regulation of CREB phosphorylation in the mouse depression model and patients with major depressive disorder^18^,^19^,^20^,^21^. Our results show that CREB phosphorylation has region dependence in the cerebral cortex of the reserpine-treated mice, which may account for the inconsistent reports. The changes in CREB phosphorylation pattern in other mouse depression model that is induced by corticosterone treatment and stress should be examined in order to determine whether the changes in CREB phosphorylation are common in depression.

An anesthetic, ketamine, quickly alleviates depression-like symptoms in mice and humans^31^. Other anesthetics affect neurotransmission including serotonin secretion^32^ whose dysregulation is considered to be a cause of depression. For these reasons, our imaging method introduced in this report, which does not require anesthetization, has advantages in the investigation of depression-related behaviors.

Principally, we cannot know how deep the source of the light exists in the brain using our method. In the previous study, we succeeded in detecting the light from hippocampus using a transgenic mouse expressing wild type luciferase^6^. However, the light intensity from split luciferase is about 100 times weaker than wild type luciferase, thus the source of light emission in current study is considered to be located in cerebral cortex. To identify the layer in which CREB is phosphorylated in the cortex, immunohistochemistry using anti-phospho-CREB antibody should be performed. To detect the light from deep region of the brain in live mouse, it will be required to use special apparatus such as optical fiber.

The fluorescence resonance energy transfer (FRET) technique is widely used for imaging protein-protein interactions in cultured cells^33^,^34^; however, it is difficult to detect FRET signals in the brain of live animal because of the huge autofluorescence in living tissues. Since split luciferase has little background noise, our method is suitable for *in vivo* imaging of CREB phosphorylation. On the other hand, it requires further improvement of brightness for imaging rapid CREB phosphorylation that occurs within a minute. Shortening exposure time by improving photon detector sensitivity or luciferin brightness^35^ may enable the observation of rapid CREB phosphorylation.

## Methods

### Ethics statement

All the experimental protocols were approved by the Animal Experiment Committee of the University of Toyama (Approval number: 2012-med-7). Animal care and experimental protocols were carried out in accordance with the Guidelines for the Care and Use of Laboratory Animals of the University of Toyama.

### Tg mice

The mouse BAC clone RP23-97O1 (BACPAC Resources Center CHORI) was modified to generate the Tg mouse line. The fragment consisting of nuclear localization signal (*NLS)-lucN-KIX*, *IRES*, and the *NLS-KID-lucC* sequence was inserted to the start codon of the *β-actin*-coding region by homologous recombination (see Supplementary Fig. S1a) using a Counter-selection BAC modification kit (Gene Bridges) in accordance with the manufacturer's instruction. The modified BAC clone was linearized using *Nru*I and then injected into fertilized one-cell embryos from C57BL/6 mice to obtain Tg mice.

### Genotyping of Tg mice

We obtained one Tg mouse line by injecting the BAC into fertilized eggs, and F2 offsprings were analyzed by southern blotting using genomic DNA from tail biopsy samples. Genomic DNA was digested with *Dra*I, electrophoresed, and transferred to a nylon membrane (Hybond N+, GE Healthcare). After the incubation with the ^32^P-labeled probe, which was derived from the promoter region of β-actin (see Supplementary Fig. S1a), the hybridized bands were detected using the BAS5000 system (Fujifilm). Some mice showed a higher intensity of the Tg-derived band (9.5 kb) than of the endogenous band (6.5 kb); these mice were regarded as homozygotes (see Supplementary Fig. S1b). The homozygous line did not show any abnormality in appearance and behavior, and the mice used in experiments were generated by mating of homozygotes.

### *In vivo* imaging

The head skin of a Tg mouse was removed and a steel plate was attached at the region posterior to the lambda and fixed with screws and dental cement on the skull five days before the first image acquisition. Luciferin (0.25 M, 300 μl) was intraperitoneally injected 5 min before the imaging. During the imaging, the steel plate was flanked by magnets (see Supplementary Fig. S2b). This procedure enables the fixing of the head without restraining other parts of the body. The mouse whose head was fixed was placed in a dark box equipped with a CCD camera with an image intensifier (Aequoria-2D/C8600 system, Hamamatsu Photonics). The number of photons emitted from the mouse head during a 5-min exposure was counted using Wasabi software. For the ROI definition, the first ROI (50 × 50 pixels) was set on the bregma, and the subsequent ROIs were set adjacently to the first ROI. For the analysis of changes in CREB phosphorylation pattern, the light intensity of a particular ROI was divided by that of all the ROIs to obtain the proportion. Then, the proportion of a particular ROI in post-treatment image acquisition was divided by that in pretreatment image acquisition to obtain the change in the intensity of light emission from the ROI.

### Western blotting

The mouse cerebral cortex was removed and homogenized using Polytron homogenizer (Kinematica) in M-PER solution (Thermo Scientific). Proteins separated by SDS-PAGE were electrically transferred onto a PVDF membrane (GE Healthcare). The membrane was incubated with, rabbit anti-phospho CREB (1:500, Bioworld) and rabbit anti-CREB (1:500, Cell Signaling Technology) in the Can Get Signal solution (Toyobo) at 4°C overnight. The membrane was reacted with a secondary goat anti-rabbit IgG HRP-conjugated antibody (1:10000, Bio Rad) and visualized using an ECL prime western blotting reagent (GE Healthcare). Chemiluminescence signals were detected using LAS-4000 mini (GE Healthcare) and quantified using Image Quant software (GE Healthcare).

### Quantification of corticosterone

The blood of the mice was taken and serum was collected from the supernatant after centrifuge (800 × g, 3 min). Quantification of corticosterone was performed using Detect X Corticosterone Enzyme Immunoassay Kit (Arbor assays) according to the manufacture's instruction.

### Quantitative PCR analysis

Total RNA was purified from the adult mouse cerebral cortex using an RNeasy mini kit (Qiagen) and reverse-transcribed using Super Script III (Invitrogen). The stratagene MX3005P system (Agilent Technologies) with the Thunderbird SYBR qPCR Mix (Toyobo) was used in the comparative quantification mode. The Ct of glyceraldehyde-3-phosphate dehydrogenase (GAPDH) was used for the calculation of the relative quantity of *lucN-KIX, KID-lucC* mRNA. The primers used were 5′-acagtccatgccatcactgc-3′ and 5′-taggaacacggaaggccatg-3′ for *Gapdh*, 5′-tcgtattcgtgagcaagaaaggg-3′ and 5′-tcacgaaggtgtacatgctttgg-3′ for *lucN-KIX*, *5*′-cagccgaactggagagcatcct-3′ and 5′-tcggtcatggttttaccgtgttcca-3′ for *KID-lucC* and 5′- ctgagattgccaatctgctg -3′and 5′- agacatctcctctgggaagc -3′ for *c-fos*.

### Immunohistochemistry

To analyze the protein localization in the brain, 10-w-old mice were anesthetized with pentobarbital and transcardially perfused with 0.1 M phosphate buffer containing 4% paraformaldehyde. The brains were removed and fixed with the same fixative at 4°C overnight then infiltrated with 30% sucrose. Brains were embedded in the OCT compound (Sakura Finetek) and sectioned at a thickness of 20 μm using a cryostat (CM1850, Leica). For the immunostaining, the sections were reacted with rabbit anti-luciferase IgG (1:100, Promega) and mouse anti-NeuN IgG (1:500, Millipore). Then the sections were incubated with goat anti-rabbit IgG conjugated with biotin (1:2000, Millipore) and goat anti-mouse IgG conjugated with Alexa488 (1:500, Invitrogen), respectively. Subsequently the signal of luciferase was amplified with Elite ABC standard kit (Vectastain) and TSA Plus cyanine 3 system (Perkin Elmer). Fluorescent images were acquired using confocal laser-scanning microscope (TCS-SP5, Leica).

### Primary neuronal culture

Cerebral cortex of transgenic mouse from embryo (E17) was dissected out in phosphate buffer saline (PBS). The tissues were treated with 0.3% trypsin in the same medium for 30 min at 37°C. After rinsing in DMEM supplemented with 10% FCS these tissues were dissociated by gentle trituration using a disposable plastic pipette. The dissociated neurons were plated on 24-well culture dish. The neurons were cultured at 37°C in 5% CO_2_/95% air and saturating humidity. At DIV 3, culture medium was exchanged to L15 (Invitrogen) containing 5 mM luciferin. The light emission before and 1 h after forskolin (10 μM) treatment was detected by Aequoria-2D/C8600 (Hamamatsu photonics).

### Tail suspension test

The tail of a mouse was wrapped with adhesive tape approximately 2 cm from its end of the tail, and the mouse was hung upside down on a hook at 30 cm height. The behaviors of the mouse were recorded for 6 min by a video camera positioned in front of the mouse. The predominant behavior in each 5 sec period of the 6-min test was determined, and the number of immobile periods was counted as the score. The higher the score, the more serious the depression-like symptoms.

## Supplementary Material

Supplementary Information Supplementary information
