# Supplementary material for: In vivo imaging of CREB phosphorylation in awake-mouse brain
Source: Sci Rep. 2015 Jun 5;5:9757. doi: 10.1038/srep09757 (PMC4456726; doi:10.1038/srep09757)
Supplement: Supplementary Information [file srep09757-s1.pdf]

Supplementary Information

In vivo imaging of CREB phosphorylation in awake-mouse brain

Tetsuya Ishimoto, Hiroki Mano and Hisashi Mori

Department of Molecular Neuroscience, Graduate School of Medicine and Pharmaceutical Sciences, University of Toyama, 2630 Sugitani, Toyama 930-0194, Japan

Address correspondence to: Tetsuya Ishimoto, PhD, University of Toyama, 2630 Sugitani, Toyama 930-0194, Japan

Tel: +81-76-434-7233, Fax: +81-76-434-5015, E-mail: ishimoto@med.u-toyama.ac.jp

Supplementary Figure S1

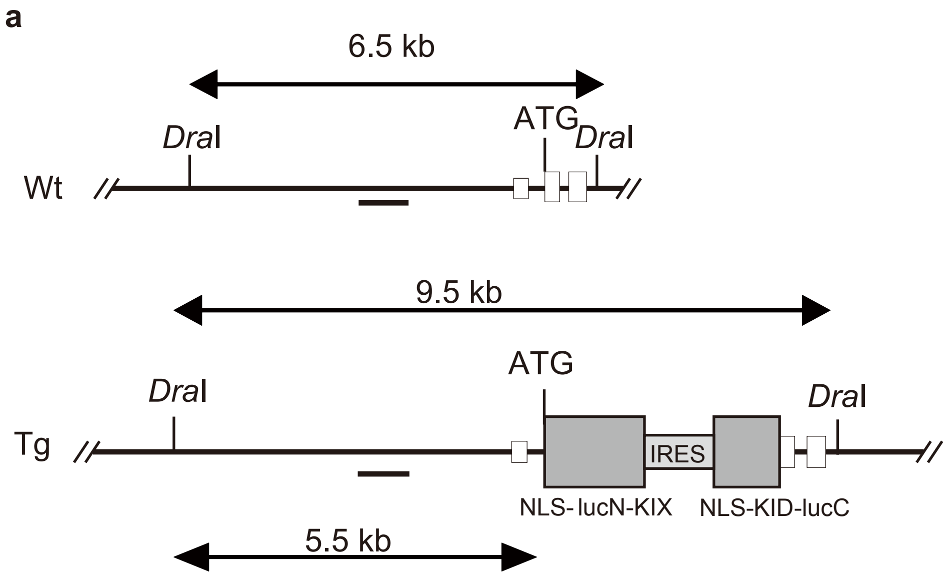

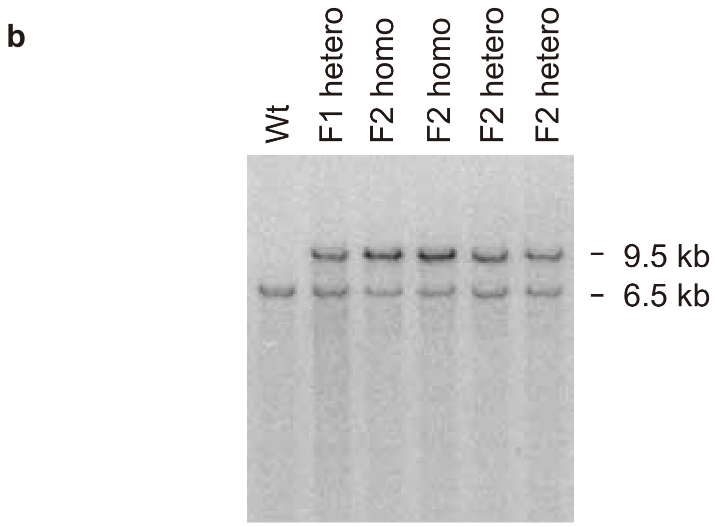

Supplementary Figure S1

Generation of Tg mouse line that expresses probe proteins.

(a) Schematic diagram of wild-type and modified BAC Tg clone mice. Large and small white boxes indicate the 5' untranslated region and open reading frame of β-actin, respectively. Gray boxes indicate the transgene. The location of the probe used in southern blotting is indicated by black bar.

(b) Southern blot analysis of Tg mice. DNA samples from tail biopsy were digested with *DraI*. Upper band (9.5 kb) signals that represent modified BAC Tg DNA are stronger in the lanes of F2 homozygous mice than in those of F1 heterozygous mice.

Supplementary Figure S2

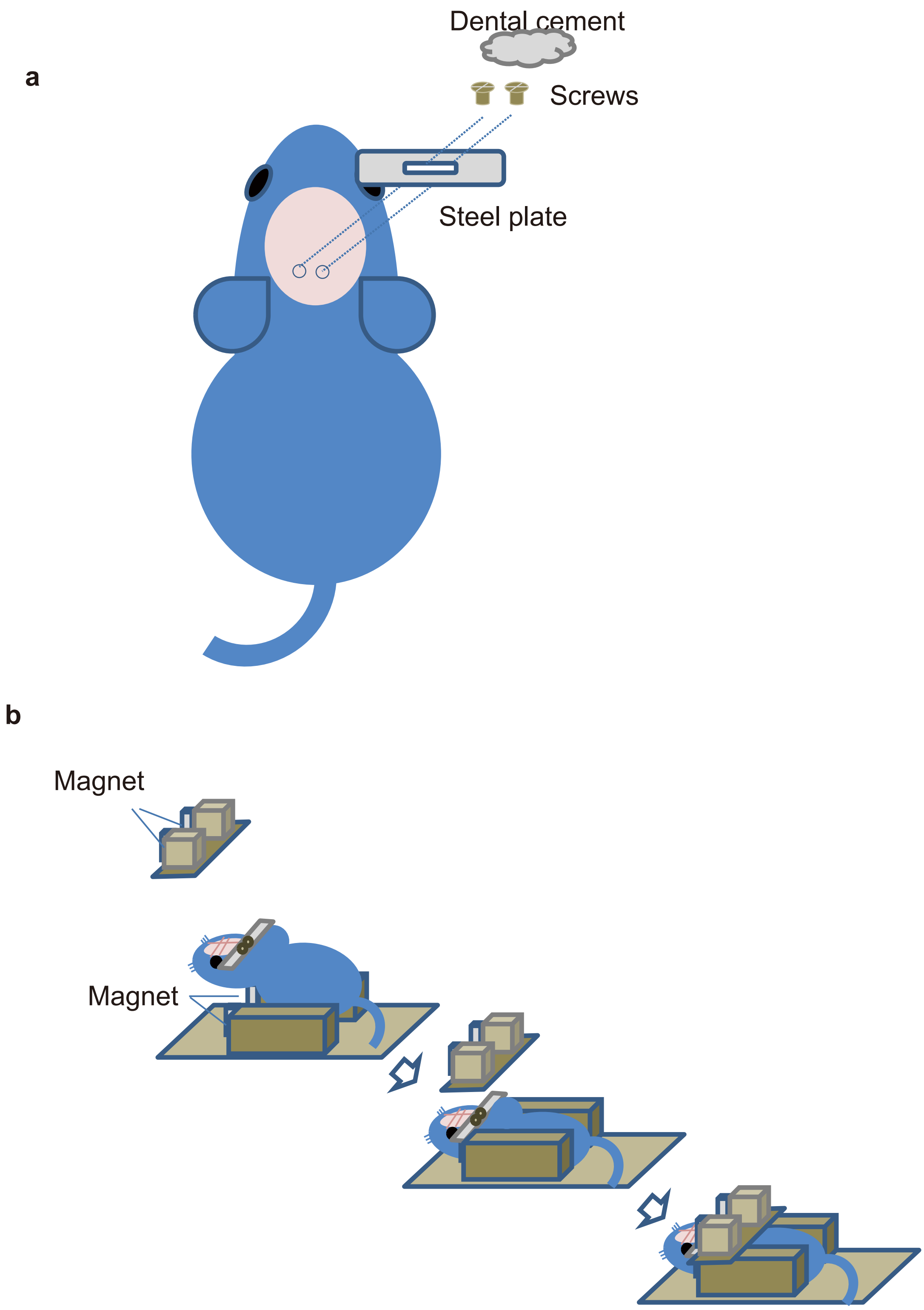

Supplementary Figure S2

Mouse fixation for in vivo imaging.

(a) Surgery for fixing the head of an awake mouse without restraining its body. A steel plate was attached to the skull at the region posterior to lambda using screws and dental cement. After the surgery, the mouse was allowed to recover for 5 days in its home cage before imaging.

(b) For the imaging of mouse cerebral cortex, the head was fixed by holding the steel plate with magnets, and the mouse was placed beneath a CCD camera in a dark box.

Supplementary Figure S3

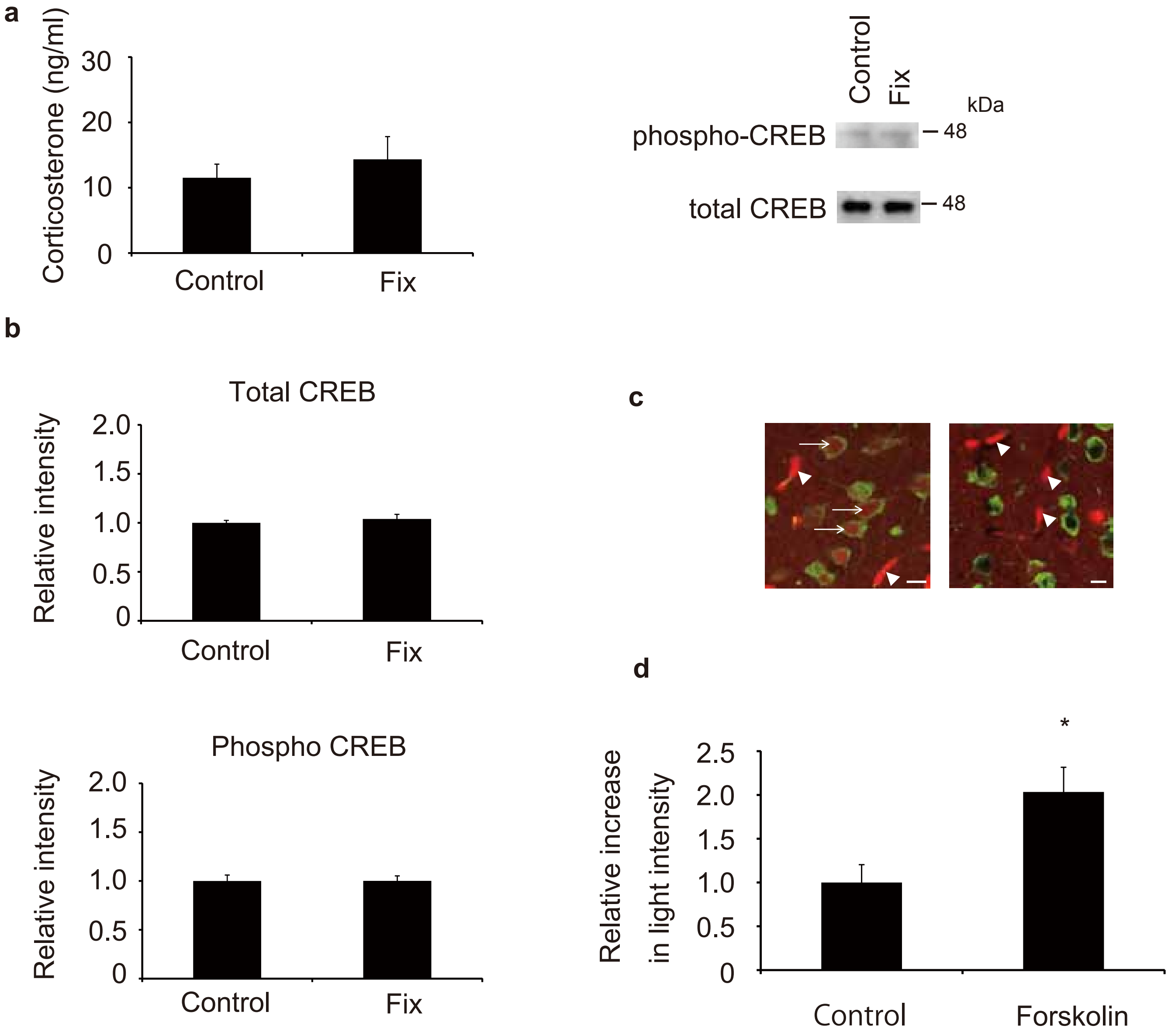

Supplementary Figure S3

(a) Changes in corticosterone concentration after mouse fixation. The serum corticosterone level after fixation are shown as mean ± SEM, n = 4.

(b) Changes in total and phosphor-CREB levels after 5 min fixation are analyzed by western blotting.

(c) Subcellular localization of the probe proteins. Slices from cerebral cortex of the transgenic mouse were reacted with anti-luciferase (red) and anti-NeuN (green) antibodies. Images are staining pattern with (left) or without (right) anti-luciferase antibody. Arrows indicate the signal of probe proteins. Arrowheads indicate non-specific staining. Bar indicates 10 μm.

(d) Forskolin-induced up-regulation of light intensity in the neuronal culture. Cortical culture from transgenic mouse was stimulated with 10 μM forskolin for 1 h. Vertical axis represents the relative up-regulation of light intensity from the neurons. Data are mean ± SEM, * $p < 0.05$, with Student' s t-test, n = 4.

Supplementary Figure S4

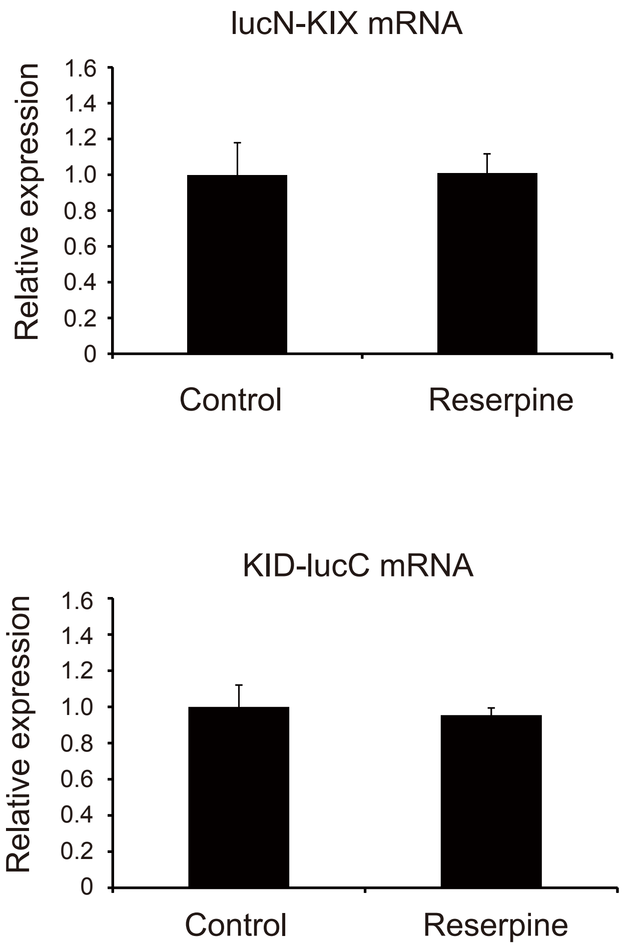

Supplementary Figure S4

Quantitative PCR analysis of mRNA expression of lucN-KIX and KID-lucC.

The mRNA expression levels in the cerebral cortex of reserpine-treated and nontreated control mice were analyzed in the comparative quantification mode. Vertical axis represents the relative expression level of transcripts to controls.

Supplementary Table S1

ROI number	Correlation coefficient between the increasing ratio of light emission and TST score
1	0.174492086
2	0.467692642
3	0.32391241
4	0.057415511
5	0.116309947
6	0.133338808
7	0.397264216
8	-0.293781998
9	-0.19911423
10	-0.086453658
11	0.201031577
12	0.477686464
13	-0.348996893
14	-0.51198964
15	-0.463236888
16	0.392220216
17	-0.40155505
18	-0.509767418
19	-0.558588539
20	-0.03659553
21	0.499571046
22	-0.216806113
23	-0.532291363
24	-0.20545448
25	-0.017730228
26	0.358974714
27	-0.365404615
28	-0.619935445
29	-0.376786296
30	0.524986454
31	-0.166452383
32	-0.648183351
33	-0.485102218

Supplementary Table 1

The coefficients of correlation between TST immobility score and changes in intensity of light emission from all ROIs are shown.
